# Nutritional Aspects of Pediatric Gastrointestinal Diseases

**DOI:** 10.3390/nu13062109

**Published:** 2021-06-19

**Authors:** Teresa Di Chio, Christiane Sokollik, Diego G. Peroni, Lara Hart, Giacomo Simonetti, Franziska Righini-Grunder, Osvaldo Borrelli

**Affiliations:** 1 Pediatric Institute of Southern Switzerland, Ospedale Regionale di Bellinzona e Valli, Via Ospedale 12, 6500 Bellinzona, Switzerland; giacomo.simonetti@eoc.ch; 2 Division of Pediatric Gastroenterology, Hepatology and Nutrition, Children’s Hospital, Inselspital, University of Bern, 3010 Bern, Switzerland; 3 Department of Clinical and Experimental Medicine, Section of Pediatrics, University of Pisa, 56126 Pisa, Italy; diego.peroni@unipi.it; 4 Division of Pediatric Gastroenterology, Hepatology and Nutrition, McMaster University, Hamilton, ON L8N 3Z5, Canada; laramhart@gmail.com; 5 Università della Svizzera Italiana, 6900 Lugano, Switzerland; 6 Division of Pediatric Gastroenterology, Hepatology and Nutrition, Lucerne Children’s Hospital, Cantonal Hospital Lucerne, 6000 Lucerne, Switzerland; 7 Division of Neurogastroenterology and Motility, Department of Pediatric Gastroenterology, University College London (UCL) Institute of Child Health and Great Ormond Street, London WC1N 3JH, UK

**Keywords:** pediatric gastrointestinal diseases, inflammatory bowel diseases, functional gastrointestinal diseases, non-IgE-mediated gastrointestinal food allergy, eosinophilic gastrointestinal diseases, diet, nutrition, food, therapy

## Abstract

In the last decade, the role of nutritional management in pediatric gastrointestinal diseases has gained increasing popularity. Disease-specific diets have been introduced as conventional treatments by international guidelines. Patients tend to more willingly accept food-based therapies than drugs because of their relatively “harmless” nature. Apart from a diet’s therapeutic role, nutritional support is crucial in maintaining growth and improving clinical outcomes in pediatric patients. Despite the absence of classical “side effects”, however, it should be emphasized that any dietary modification might have negative consequences on children’s growth and development. Hence, expert supervision is always advised, in order to support adequate nutritional requirements. Unfortunately, the media provide an inaccurate perception of the role of diet for gastrointestinal diseases, leading to misconceptions by patients or their caregivers that tends to overestimate the beneficial role of diets and underestimate the potential adverse effects. Moreover, not only patients, but also healthcare professionals, have a number of misconceptions about the nutritional benefits of diet modification on gastrointestinal diseases. The aim of this review is to highlight the role of diet in pediatric gastrointestinal diseases, to detect misconceptions and to give a practical guide for physicians on the basis of current scientific evidence.

## 1. Introduction

Since ancient Greek and Roman times, the term “disease” was literally used to denote dis-ease, physical imbalance. To maintain or to restore this balance, food and diet have always been considered crucial. The word “diet” itself comes from the Greek word “δῐ́αιτᾰ”(díaita) and latin “diaeta”, meaning “way of living”. In other words, since its etymology, the word “diet” has an intrinsic meaning of influencing, governing, or arbitrating someone’s health status through food and lifestyle.

This assumption has found scientific explanation over the centuries. It is widely recognized that diet can modify the health status by different mechanisms, such as by disrupting intestinal barrier and altering the microbiome [1].

Due to its role in maintaining the human body in balance, a change in food habits can be both beneficial but also detrimental. For instance, protein, fats, carbohydrates and probiotics cause a modification in the proportion of “potentially healthy and/or unhealthy bacteria”, which in turn might lead to immunologic and metabolic changes [2]. Mass media, such as TV and social media, influence people’s beliefs. Food advertising has a great influence on people’s food habits and patients often go on a diet by themselves without medical advice.

Apart from the role of diet in preventing diseases, it is now recognized that specific diets can be used as a treatment option for gastrointestinal diseases. In the pediatric setting, this topic has gained significant interest in the last few years. In some conditions, such as Crohn’s disease, specific diets such as exclusive enteral nutrition (EEN) have been successfully used in inducing disease remission [3,4,5]. On the other hand, for other diseases such as functional gastrointestinal disorders (FGID), the role of specific diets is still controversial [6,7]. Therefore, physicians should prescribe dietary changes with caution because, apart from its potential effectiveness as all traditional medical therapy, nutrition support in children has an added and crucial role in maintaining growth and improving clinical outcomes. However, as all therapies, dietary changes can be also harmful.

In this review, we give an overview of advances and recent evidence linking nutrition to pediatric gastrointestinal diseases. Unresolved questions, evidence-based recommendations and false beliefs will be explored for each topic. With the term “false beliefs” in each chapter, the authors refer to anecdotal notions, frequently reported by parents, patients or mass media, which were contradicted by current evidence. Because of the vastness of the topic, we focused on a sub-group of pediatric gastrointestinal diseases: inflammatory bowel diseases, functional gastrointestinal diseases, non-IgE-mediated gastrointestinal food allergies, eosinophilic gastrointestinal diseases. 

## 2. Materials and Methods

A search for suitable studies was performed using scientific databases (PubMed, Embase, Medline, Cochrane library, Web of Science databases). The search was performed separately by each co-author accordingly to each topic. The subsequent Medical Subject Headings (Mesh) were used by all authors: “Nutrition” [Mesh], “Diet” [Mesh], “Food” [Mesh], “Therapy [Mesh]”. The following topic-specific Mesh were used along with the previously mentioned Mesh by each author accordingly to each sub-chapter’s topic: “Inflammatory Bowel Disease” [Mesh], “Functional Gastrointestinal disease” [Mesh], “Non-IgE-mediated Gastrointestinal Food Allergy” [Mesh] or “Eosinophilic Gastrointestinal Diseases” [Mesh], respectively for the Section 3.1, Section 3.2, Section 3.3 and Section 3.4. Both “AND” and “OR” as Boolean operators were used in order to be as exhaustive as possible. Further studies were searched using the bibliography in articles collected from the searches. Only the most relevant manuscripts were included. 

## 3. Nutritional Aspects of Pediatric Gastrointestinal Diseases

### 3.1. Inflammatory Bowel Disease and Diet

(1) Exclusive enteral nutrition.
  Evidence-based facts:
    - Exclusive enteral nutrition (EEN) is as effective as steroids in induction of remission in pediatric Crohn’s disease patients.
  False beliefs:
    - Exclusive enteral nutrition has no role in complicated Crohn’s disease (CD).

Already 40 years ago the benefit of EEN for pediatric Crohn’s disease (CD) patients could be demonstrated [8]. EEN is not only as effective as corticosteroids in inducing remission in pediatric CD, but also mucosal healing is significantly more likely with EEN than with corticosteroids [3,4,5]. EEN consequently has been established as crucial steroid-sparing strategy in pediatrics and is recommended as first-line therapy in pediatric luminal CD in international guidelines [9,10]. Lately, biological therapy has been used more often in top-down strategies and earlier in the disease course even in pediatric patients [11,12]. Especially in pediatric patients with extensive disease or strictures, biological therapy is recommended as first-line induction therapy [10]. However, one pediatric study suggested that EEN is as effective as biological therapy in achieving clinical remission in these cases [11]. Additionally, success rate of EEN seems to be independent of disease location and should therefore not restrict the decision to use EEN [13]. Case reports have shown the beneficial role of EEN even in pediatric patients with perianal disease [14]. In adults with complicated disease, including intestinal fistula/abdominal abscess or inflammatory strictures, EEN led to clinical remission, fistula closure, resolution of abscesses, or improvement of strictures in a significant number of patients [15,16]. In line with this, it was shown that EEN could be used to reduce operative risks, and in some cases avoid the need for surgery in structuring or penetrating disease. [17,18,19]. 

There is still discussion about the mechanisms by which EEN induces remission. Especially in CD, dysbiosis with a decreased bacterial diversity and an altered abundance of different taxa is postulated as a key factor [20,21]. Indeed, microbiome analysis shows a shift in microbial composition during EEN therapy [4,22,23]. However, the changes in the microbiome are not consistent between studies and no specific profile reflecting disease control has been identified so far. As another mechanism of action, a direct anti-inflammatory effect of EEN has been suggested. In in vitro models a positive effect of EEN on inflammatory responses of cultured cells as well as decreased permeability and improved tight junction stability was shown [24,25]. Fitting these findings, mucosal cytokine analysis in patient samples before and after EEN demonstrated a down-regulation in proinflammatory cytokines [26,27]. However, the components of EEN able to induce mucosal healing have not yet been identified.

(2) Partial exclusive enteral nutrition and other dietetic strategies.
  Evidence-based facts:
    - Partial exclusive enteral nutrition is less effective than EEN.
  False beliefs:
    - Ordinary solid food-based diets have no value in IBD.

There are multiple newly developed diet regimens created with the aim to overcome poor compliance to EEN. These diets involve combining EEN formulas with specific solid food-based diets, reproducing EEN by means of solid food [28], or avoiding specific food elements such as carbohydrates [29,30]. These dietary approaches are based on clinical observations and pathogenic mechanisms involved in IBD [31]. They aim to positively influence the microbiome and restore the intestinal barrier and the intestinal immune homeostasis by regulating components of the diet. The Crohn’s disease exclusion diet (CDED) includes 5 mandatory daily foods (chicken, eggs, bananas, apples, and potatoes) and avoids or reduces certain fats, processed meats, emulsifiers, artificial sweeteners, carrageen, and sulfites [32]. The Crohn’s disease treatment-with-eating diet (CD-TREAT) restricts dietary components like gluten and lactose and matches carbohydrates and proteins to EEN [28]. The specific carbohydrate diet (SCD) eliminates grains, as well as most sugars, milk products, and processed foods [30]. Not surprisingly, these diets were better tolerated than EEN, and were effective in achieving a clinical response or inducing remission in mild to moderate CD [28,29,32]. However, there needs to be significant family education and counseling by a dietitian, which requires these resources be available in order for the diet changes to be effective. 

Partial enteral nutrition (PEN) combined with a normal diet may be easier to implement but is less effective. In comparison to EEN, PEN improves clinical symptoms but induced remission less often [11,33]. In general, PEN has shown benefit mainly as adjuvant therapy that can prolong remission after successful EEN [34] or medical therapy [35], and as a tool for improving the child’s nutritional status [36,37]. The combination of PEN with medical therapy is an interesting option for more severe disease phenotypes, e.g., in patients requiring a dose escalation due to loss of response to a biologic. This option is associated with a higher rate of clinical remission than in medical-only therapy [38]. Other dietary interventions explored in adults such as a diet low in red and processed meat [39] or a gluten-free diet [40] did not show a benefit in preventing relapses in CD patients.

(3) Prebiotics and probiotics and other food supplements/additives.
  Evidence-based facts:
    - Currently, pre- and probiotics have not produced a resounding success in IBD therapy.
  False beliefs:
    - There is no value in further investigating specific microbiota-targeting therapeutic options by means of pre- and probiotics.

Dietary fibers are non-digestible carbohydrates that are not hydrolyzed or absorbed in the small intestine of humans. In the colon, soluble fibers are fermented by bacteria, which thereby produce beneficial short-chain fatty acids. Adult studies of CD patients have demonstrated alterations in the microbiome composition and a clinical benefit after prebiotic supplementation with *Bifidobacterium longum* [41]. The SCFA butyrate was lately identified as a possible mediator of the healing effect of anti-TNF-alpha therapy in IBD [42]. Following anti-TNF-alpha therapy, the microbiota profile shifted towards a healthy one and increased levels of butyrate and substrates involved in butyrate synthesis were significantly associated with clinical remission [42]. These may suggest that increase of butyrate with fiber therapy is insufficient, whereas anti-TNF-alpha therapy induces enough production for a beneficial effect. Therefore, butyrate supplementation may be a treatment approach worth exploring [43].

Probiotics can directly affect the intestinal microbiome and therefore can potentially correct the dysbiosis found in IBD. Interestingly, positive effects of probiotic supplementations are mainly seen in UC, whereas data in CD so far are discouraging [44,45]. VSL#3, a multi-strain mix, induces remission in mild to moderate UC [46] and has positive effects on disease activity as adjunct therapy [47]. However, studies using probiotics are prone to multiple biases and thus generate results with low-grade evidence [48].

Omega-3 fatty acids contained in fish oil have anti-inflammatory characteristics and therefore multiple studies have examined their effects in prolongation of remission in IBD. However, a Cochrane Review found that Omega-3 fatty acids are likely to be ineffective in maintenance of remission [49].

Most studies investigating dietary interventions or food supplementations and additives focus on CD. Yet, curcumin, a food spice with anti-inflammatory and antioxidant effects, has shown potential as an effective adjunctive therapy in adult and pediatric UC patients [50,51].

(4) Malnutrition.
  Evidence-based facts:
    - Treatment/prevention of malnutrition is advised in pediatric IBD patients.
  False beliefs:
    - Only CD patients are at risk of malnutrition.

The causes for malnutrition include (i) reduced oral food intake due to abdominal pain and diarrhea associated with food consumption, (ii) malabsorption and intestinal loss of nutrients, (iii) increased energy requirements due to systemic inflammation, (iv) iatrogenic factors due to treatment, and (v) misconception of the nutritional status. Children with IBD are especially vulnerable to and affected by malnutrition, and this can have significant implications for their physical and pubertal development [52,53]. One subsequent effect of malnutrition is the relatively high prevalence of sarcopenia in pediatric CD patients [54]. However, sarcopenia is not limited to CD patients and can be found in UC as well [54]. In the long run, malnutrition leads to shorter final adult height, especially if Crohn’s disease occurs early in childhood [55,56]. Another consequence is an altered body compositions with a higher rate of myopenic and myopenic-obese patients in the IBD group [57]. 

Iron and vitamin D deficiencies are common among pediatric CD and UC patients [58,59] and often persist during follow-up [60]. In general, screening for vitamin and trace element deficiencies are recommended in patients at risk of malnutrition and in patients on exclusion diets for long periods [61]. Screening bloodwork should be included in the follow-up of IBD patients with the aim of normal hemoglobin and replenished iron stores [62].

*Inflammatory Bowel Diseases: Conclusion*

A large number of environmental factors (antibiotics, smoking, Vitamin D, infections, delivery modus, previous therapies) are influencing the disease course in IBD. Diet and nutrition can be considered among the environmental factors. However, as a special diet does not cause IBD, dietary restrictions cannot heal IBD either. Nonetheless, there is therapeutic value in including dietary changes in treatment considerations. EEN and other specific diets have been shown to be as effective as medical therapy in inducing and maintaining remission in pediatric IBD patients. They should be further explored as an optimal low-risk high-benefit treatment strategy. 

### 3.2. Functional Gastrointestinal Diseases

#### 3.2.1. Functional Defecation Disorders (Infant Functional Diarrhea and Dyschezia) and Diet

(1) Fluids and fibers.
  Evidence-based facts:
    - Increase of fluid or fiber intake to recommended weight and age-adapted levels is essential.
  False beliefs:
    - Excessive increase of fluid or fiber intake is helpful in treating children with functional constipation.

A meta-analysis from Yang et al., including one adult and four pediatric double-blind studies, revealed that additional fibers can increase stool frequency in functional constipation. However, there was no notable effect in overall treatment success [63]. Nevertheless, fluid and fiber intake at a normal, recommended level for age and weight is essential, especially in children with functional constipation [64,65,66,67].

(2) Probiotics and prebiotics.
  Evidence-based dietary recommendations:
    - Probiotics and prebiotics might positively influence the defecation behaviors but without proven evidence of superior treatment effect compared to placebo or standard treatment in childhood.
  False beliefs:
    - Probiotics and prebiotics are superior to placebo effect in childhood defecation disorders.
(2a) Probiotics.

Several RCTs are available in childhood functional constipation comparing probiotics to placebo or to osmotic active substances such as lactulose or polyethylene glycol (PEG) [68,69]. *Lactobacillus rhamnosus* GG as adjunctive therapy to lactulose and lactobacillus casei rhamnosus Lcr35 did not show a superior effect compared to placebo [70]. Coccorullo et al. compared *Lactobacillus reuteri* DSM 17938 with placebo in an eight-week intervention trial in infants. An increase in stool frequency at weeks two, four, and eight was found, but stool consistency and episodes of inconsolable crying did not differ between the groups [71]. *Lactobacillus reuteri* DSM 17938 combined with macrogol compared to macrogol alone and matching placebo did not demonstrate significant differences in defecation frequency and stool consistency, painful evacuation, fecal soiling, and abdominal pain [72]. Other RCTs investigated the effect of *Bifidobacteria*, added in goat milk yogurt vs. goat milk yogurt without additional probiotics or *Bifidobacteria* added to polyethylene glycol (PEG) 4000 vs. PEG alone on symptom improvement in functional constipation, without a significant superiority to control treatment [73,74]. However, some beneficial effect of *Bifiodobacteria* (increase of stool frequency, decrease in fecal incontinence) in childhood functional constipation might be present, as shown by a pilot study of Tabbers et al. [75].

(2b) Prebiotics.

Prebiotics are non-digestible short-chain carbohydrates that influence the growth and activity of gut microbiota. In infants, several studies showed beneficial effect of oligosaccharide supplementation in infant formula [76,77]. However, the evidence is too low to recommend prebiotics as a treatment in defecation disorders in infants [65,66,78].

No RCTs are so far available in children older than one year. 

The current treatment guidelines of the North American and European society of pediatric gastroenterology do not recommend the use of either probiotics or prebiotics in children with functional constipation [66].

(3) Partial/extensive hydrolyzed formula or alternative cow milk (CM)-free diets.
  Evidence-based dietary facts:
    - In therapy-refractory chronic constipation, especially in infancy, a trial of a two-to-four-week period with partial hydrolyzed formula (pHF) or CM-free diet can be considered.
  False beliefs:
    - Children with chronic functional constipation have a higher risk of CM allergy.

In infants and young children, an association of CM allergy and constipation was discussed in the 1990s [79]. Iacono et al. performed a double-blind crossover study comparing CM with soy milk in 65 patients age 11 to 72 months with chronic constipation. The response rate to soy milk was 68% versus 0% in the CM group regarding symptoms related to constipation [80]. A more recent trial in children showed similar results [81]. A double-blind, randomized crossover study using a mixture of pHF combined with prebiotic oligosaccharides (sn-2 palmitate) in 38 constipated infants, 3- to 20-weeks-old, showed improvement of stool consistency, but not of frequency [77]. Fatty acids at position sn-2 are likely to have a stool-softening effect [82]. Borrelli et al. investigated for potential underlying mechanisms in children with both food allergy and functional constipation. They found a significant decrease of mast cells and decrease of anal resting and residual pressure in the group of constipated children responsive to oligoantigenic diet, suggesting that in constipated children with food allergies, anal motor function and mast cell density might be affected positively by oligoantigenic diet [83].

In childhood, one clinical randomized trial compared constipated children on CM diet (CMD) with children on CM-free diet (CMFD). PEG solution 0.5 g/kg/d was administered in both groups. Of all children in the CMFD group, 80% showed improvement of constipation vs. only 47.1% in the CMD group (*p* = 0.0001). Furthermore, after a two-week reintroduction phase of CM in the CMFD group, symptoms of constipation worsened again [84].

There is little evidence that a lactose-reduced milk might have a positive impact on stool frequency and consistency [85]. However, as many pHF or extensive hydrolyzed formula also have reduced lactose content, this might be an interesting point for further investigation.

Overall, there are no randomized placebo-controlled studies assessing the efficacy of pHF or extensive hydrolysates as single intervention in constipated infants and children [78]. No data exist to support special diets in infantile dyschezia. 

In conclusion, actual guidelines and consensus statements based on expert opinion are recommending a CM free or pHF formula for two to four weeks only in the presence of therapy-refractory functional constipation in non-breastfed infants [66,82].

#### 3.2.2. Functional Abdominal Pain Disorders and Infantile Colic and Diet

(1) Probiotics, prebiotics, and fibers.
  Evidence-based dietary facts:
    - Lactobacillus reuteri DSM17938 showed beneficial effect in exclusively breastfed babies with infantile colic.
(1a) Probiotics.

In infants with colic, one hypothesis is that there is a lower diversity and stability of the microbiome comparing to infants without colic. This supports the idea of an underlying dysbiosis leading to a modulation of the gut–brain axis [86]. Therefore, it is not surprising that several trials showed benefits of probiotics and prebiotics in this patient group, with a decrease of crying time in babies, breast- or formula-fed. The best-studied bacteria species is *Lactobacillus reuteri* DSM 17938 [87,88,89,90,91]. A recent meta-analysis by Sung et al., including four double-blind randomized trials of *L. reuteri* DSM17938 versus a placebo, revealed a significant intervention effect of probiotics in breastfed, but not in formula-fed infants [92]. The effect of *Lactobacillus rhamnosus* in infant colic was studied by Pärtty et al. in children already undertaking a CM exclusion diet, and it showed no difference compared to the placebo group. However, in both groups, the daily crying time was reduced compared to baseline, suggesting that CM exclusion diet might explain the findings [93]. 

A Cochrane review in 2019 concluded that probiotics are not more efficacious than placebo in the prevention of colic in infants, but daily crying time might be significantly reduced [94]. Probiotics are demonstrated to be superior to placebo in decreasing crying time also in recent meta-analyses [95,96].

In children with functional abdominal pain disorders, such as functional dyspepsia, functional abdominal pain (FAP), and irritable bowel syndrome (IBS), there is minimal evidence supporting the beneficial role of probiotics. The existence of positive effects of probiotics to decrease pain intensity and frequency in FAP is questionable [97,98,99,100,101].

(1b) Prebiotics and fibers.

Dietary fibers are non-digestible carbohydrate polymers, fermented in the colon by the gut bacteria. They also stimulate microbial growth, leading to an increase in microbial mass.

Prebiotics are non-digestible, selectively fermented ingredients, that influence the composition and activity of the gut microbiota acting on the gastrointestinal microbiome and in pediatric gastrointestinal disorders [102]. 

In patients with infantile colic, an increase in Gram-negative bacteria and a lower diversity of the microbiome was described. Therefore, adding fibers and prebiotics might play an important role in treatment. Treem et al. compared soy fibers to placebo, but did not find any significant effect on crying or fussing time [103]. A formula with fructooligosaccharides and galactooligosaccharides and added simethicone was studied in a randomized controlled trial showing a significant reduction of symptoms compared to controls [104]. The study by Vandenplas et al. demonstrated that fermented infant formula with short-chain galacto-oligosaccharides and long-chain fructo- oligosaccharides decreases the incidence of colic in infants [105]. A positive effect of prebiotics and probiotics was also found in a study by Pärtty et al. [106].

In children with FAP and irritable bowel syndrome (IBS), there is a reduction in beneficial bacteria such as *Bifidobacteria*, and an increase in opportunistic bacteria such as proteobacteria. This leads to a potentially dysbiosis and is considered a contributing factor in pathogenesis in FAP and IBS [102].

Feldman et al. and Christensen performed the first randomized controlled trials in pediatric patients with recurrent abdominal pain. Feldman’s group studied the effect of corn fiber vs. placebo, while Christensen’s group assessed ispaghula husk vs. placebo [107,108]. Corn fiber showed a significant reduction in the number of abdominal pain episodes, but ispaghula showed no additional benefit compared to placebo. In more recent trials, partially hydrolyzed guar gum compared to placebo showed a clinical improvement of symptoms in children with IBS, glucomannan vs. placebo did not show any differences in children with FAP, and psyllium fiber compared to placebo in children with IBS had a significant effect on reducing pain episodes [109,110,111].

In conclusion, probiotics might have a positive effect in reducing daily crying time in breastfed babies with infantile colic (*L. reuteri* DSM17938), and in reducing symptoms in children with IBS (*L. rhamno* sus GG) and FAP (*L. reuteri*). Prebiotics and fibers might be considered as additional treatments in infants with colic and in children with FAP and IBS.

(2) Carbohydrates and diet low in fermentables oligosaccharides, disaccharides, monosaccharides, and polyols (FODMAP).
  Evidence-based dietary facts:
    - A reduced FODMAP diet can be a therapeutic option in a subgroup of children with functional abdominal pain disorders, namely IBS.
  False beliefs:
    - FODMAP diet is an established diet recommended for functional abdominal pain in pediatric patients.

Carbohydrates consist of monosaccharides and disaccharides, sugar alcohols (polyols), and oligosaccharides [112]. The causal role of carbohydrate malabsorption in functional abdominal pain in childhood and the diagnostic benefit of hydrogen breath test (H_2_BT) have not been proven. Several older trials showed no beneficial effect of lactose restriction in pediatric patients with functional abdominal pain [113,114]. There is not enough evidence to support the hypothesis that lactose intolerance or fructose malabsorption might be responsible for functional chronic abdominal pain in childhood [115]. In a German cohort of 253 children with functional abdominal pain, H_2_BT was performed in 135 children who reported abdominal pain on lactose or fructose consumption. Carbohydrate malabsorption was found in 41% by H_2_BT, however, after removing the offending foods, only 18% had improvement in abdominal pain [116]. Another study showed an improvement of symptoms on a low-fructose diet in 103 pediatric patients with frequent abdominal pain, but no association with a positive breath test was seen [117].

FODMAP is a diet low in fermentable oligosaccharides, disaccharides, monosaccharides, and polyols. In adults, this diet is indicated as a second line treatment in IBS [118,119]. In breastfeeding mothers, low-FODMAP diet might decrease crying-fussing periods in babies with colic, but the evidence was not substantial [120].

A recent randomized controlled trial in 22 children on a low-FODMAP diet for functional abdominal pain showed no significant improvement in comparison to children on a normal unrestricted diet [6]. Furthermore, a low-FODMAP diet in children is difficult for parents to follow and maintain, which might influence the effectiveness and acceptability of this particular diet in children [121]. A recent Cochrane analysis from 2017 about diets in recurrent abdominal pain in children revealed only one controlled, double-blind randomized clinical using low-FODMAP diet [7]. In this trial, 52 children aged 7 to 17 years old with IBS were randomized to either a typical American childhood diet (TACD) or a low-FODMAP diet, with a crossover during the study. Thirty-three children completed the study. Children on a low-FODMAP diet had significantly fewer pain episodes compared to their baseline and to children on TACD [122].

The same group (Chumpitazi et al.) conducted a double-blind placebo-controlled crossover trial in children with IBS in 2018, comparing fructans with placebo (maltodextrin). They identified a subgroup of children with increased abdominal pain frequency when challenged with the FODMAP carbohydrates fructans. However, the study could not prove a causality between fructans intake and IBS in this cohort [123].

In summary, a low-carbohydrate or low-FODMAP diet in children should not be considered as first-line treatment in functional abdominal pain disorders due to insufficient supporting evidence. However, in individual cases it could be considered as an additional or alternative treatment in children with IBS [124].

(3) Cow’s milk, partial/extensive hydrolyzed formula, or alternative cow’s milk-free diets.
  Evidence-based facts:
    - The role of CM protein allergy as a contributing factor to the pathophysiology of functional abdominal pain disorders in pediatric patients remains unclear. However, patients with food allergy triggered by CM might experience functional abdominal pain.
  False beliefs:
    - CM allergy has to be considered as a causative trigger of functional abdominal pain in children.

Cow’s milk is discussed as a potential factor contributing to the pathophysiology of infantile colic, as it is one of the first food proteins that infants are exposed to [86]. Positive effects of CM protein-free diet in breastfeeding mothers was observed in at least one-third of infants in one study [125]. In this study, a double-blind crossover trial with whey protein capsule was later performed: nine out of the ten infants reacted with colic after this challenge [125].

A double-blind clinical trial compared standard CM formula to soy milk or extensive hydrolysate, with a reduction of colic symptoms observed in 68% of infants in the intervention group [126]. Comparable positive results were observed in a more recent double-blind randomized and placebo-controlled trial, where a whey hydrolysate was superior to standard formula [127]. Casein hydrolysate also appeared to be superior to normal formula in reducing colic symptoms in infants [128]. A systematic review in 2012, including 12 randomized controlled trials, further provided evidence that maternal elimination diet (CM protein-free) in breastfed infants and hydrolyzed formula in formula-fed infants can significantly reduce symptoms in babies with colic [129]. However, it is not excluded that this positive effect was because of treated CM protein allergy (CMPA), as colicky symptoms can be also due to CMPA.

However, most of the clinical trials included only short-time interventional intervals and did not include a reintroduction phase of CM. A conclusion about long-term effect is therefore not possible.

The role of CM protein intolerance (non-IgE hypersensitivity) or allergy (IgE-related CMPA) as a contributing factor to the pathophysiology of functional abdominal pain disorders in childhood remains unclear. A recent review by Pensabene et al. including a pubmed and Cochrane Database research was published in 2018 [130]. There was a long discussion about whether CMPA might cause FAP and IBS, with no clear evidence so far. One of the main challenges is the occurrence of gastrointestinal symptoms soon after ingestions of CM protein-containing meals, assuming a direct association with the ingested food. When focusing on pathophysiology, CMPA in infancy might act as an early inflammatory life event, leading to visceral hypersensitivity later during childhood leading to functional abdominal pain disorder.

In conclusion, alternative diets to CM, such as CM elimination diet in breastfeeding mothers or hydrolysates in infants, might be considered in infant colic. No evidence exists so far to recommend CM elimination diet with functional abdominal pain disorders.

(4) Gluten.
  Evidence-based facts:
    - Evidence about the role of gluten exposure as a triggering factor in FAP in children is lacking.
  False beliefs:
    - Gluten sensitivity in absence of celiac disease is not associated with IBS.

A relationship between functional abdominal pain disorders, namely IBS and gluten intake, is described in adult studies. Reduction of wheat in adults with IBS showed a positive effect with significant symptom reduction after using this exclusion diet. Studies in children are lacking. Regarding the potential pathophysiological effect of gluten to produce pain signals, it was found that the degraded gluten protein in the gut might lead to gut immunity and/or functional abnormalities in a subgroup of patients with IBS. Furthermore, wheat contains fructans and other fermentable oligosaccharides, disaccharides, mono-saccharides, and polyols (FODMAPs). It may therefore not be the gluten, but the poorly absorbed carbohydrates in the intestine that lead to abdominal pain. The recent review study by Llanos-Chea et al. nicely summarizes the role of gluten in functional abdominal pain disorders in childhood [131]. In conclusion, no recommendation of a gluten restriction can be made in pediatric patients with FAP.

*Functional gastrointestinal disorders: Conclusion*

FGIDs in children might be influenced by what children are eating. The microbiome contributes to the pathophysiologic mechanism of FGIDs, and it is therefore not surprising that nutrition plays an important role as well. However, it is still unclear and controversial as to what the optimal diet should be for these functional conditions in infants and children. Good evidence is frequently missing in most of the abovementioned diet modification studies. Changes to diet and nutrition should therefore be discussed on an individual basis. 

### 3.3. Non-IgE-Mediated Gastrointestinal Food Allergy

#### 3.3.1. Food Protein-Induced Enterocolitis Syndrome (FPIES) and Diet

Evidence-based facts:
  - CM- and soy-triggered FPIES are more frequent at 3–6 months of life; Solid foods-triggered FPIES begin at 4–7 months;
  - Because of the frequent co-reaction between CM and soy, soy formula should be offered under medical guidance in patients with CM-triggered FPIES and unknown soy tolerance, and vice versa. Patients with CM-triggered FPIES can tolerate donkey and/or camel milk;
  - Most children become tolerant to the triggering food by 3–5 years of age.
False beliefs:
  - Patients with CM-triggered FPIES can have goat and sheep milk;
  - Dietary elimination of offending triggers in breastfeeding mothers should be recommended routinely, even if the child is thriving and is asymptomatic.

FPIES triggers differ depending on geographic areas, presumably reflecting diverse eating customs in each area. 

In the first 3–6 months of life, FPIES is mainly triggered by CM and soy, with food introduction from 4 to 7 months this shifts to solid foods (e.g., rice, oats, eggs, fish) [132,133]. 

Between 65% and 68% of children react to one food antigen, 88–91% of the children have reactions to one or two foods, rarely more than two offending foods are identified [134,135]. Data on triggering foods’ co-reactivity and food tolerance are summarized in Table 1. Overall, after the first year of life, new-onset FPIES is very uncommon [134,136]. FPIES is a dose-dependent reaction, depending on how often and how much of an incriminating food is ingested. 

Children with CM- and soy- triggered FPIES should receive casein-based extensively hydrolyzed formula as the initial therapeutic step; however, 10% and 20% of these patients may require an amino acid-based formula [137,138,139,140]. 

Fish- and shellfish-induced FPIES are most commonly reported in littoral areas such as Spain [141,142,143] or Italy [144] where these foods are introduced earlier into the infants’ diet [143,145,146]. Sole and cod were the most frequently identified offending fish [141,142,146]. 

After a prolonged phase of avoidance of the triggering food, patients can develop secondary IgE-mediated allergy when the food is reintroduced. This can be evaluated with skin prick tests for that specific food [134,144,147].

A strict dietary elimination for a prolonged time should be done under the guidance of a dietician or nutritionist, regardless of the number of foods eliminated [140]. While CM-/soy-triggered FPIES tolerance is reached for most in the first year of life, other food FPIES tolerance occurs later: e.g., 3 years of age for grains and 3.5 years for other solid foods [140]. Food aversion and delay in the acquisition of feeding competencies have been described in patients on long-term dietary restrictions. Therefore, introduction of different tastes and textures should be recommended to families in order to improve flavor acceptance and reduce the risk of atypical eating behaviors [148].

#### 3.3.2. Food Protein-Induced Allergic Proctocolitis (FPIAP) and Diet

Evidence-based facts:
  - The first therapeutic step is eliminating CM from the infant and mother diet (if breastfeeding),
False beliefs:
  - Breastfeeding can in all cases be continued;
  - Symptoms of IgE-mediated food allergy can occur once the offending food is re-introduced after restricted diet.
Controversial:
  - In contrast to the early elimination diet protocol, a “wait and see” approach during the first month of rectal bleeding has been proposed.

FPIAP can be triggered by either food in maternal diet (CM, soy, egg, wheat) passed into breast milk or by CM protein in infant formulas [152].

CM is the most frequently cited allergen [153]. European guidelines suggest strict CM protein-free maternal diet for 2–4 weeks. If symptoms resolve, a food challenge (clinical evaluation of the effects of food’s reintroduction) should be conducted to confirm the diagnosis [154]. If symptoms persist despite the CM-free diet, next steps include eliminating soy, followed by egg and then other suspected foods from the maternal diet. In most cases, breastfeeding can be continued [155]. In 12% of infants, symptoms persist despite modifications in maternal diet. In these cases, a universal agreement on treatment approach has not been reached and each case must be discussed individually. Options include choosing to proceed with breastfeeding irrespective of bleeding, or replacing breastfeeding with a hydrolyzed or aminoacidic formula [156]. The latter is the more often recommended treatment plan. Approximately 10% of infants end up needing aminoacidic formula, as symptoms persist even with extensively hydrolyzed options [157]. In contrast with FPIES, an IgE-mediated food allergy usually does not occur when the eliminated protein is later administered.

In contrast to the early elimination diet protocol, some authors have recently proposed a “wait and see” approach during the first month of rectal bleeding, considering that those episodes in infants are often self-resolving [156,158]. Multiples studies have reported symptom improvement without maternal dietary modifications in up to 20% of breastfed infants [156,159].

#### 3.3.3. Food Protein-Induced Enteropathy (FPE) and Diet

Evidence-based facts:
  - As CM is the most frequent trigger, the first therapeutic step should be using hydrolyzed formulas or, in refractory disease, an amino acid-based formula.

Among FPE triggered by a single food, CM is the most common culprit. Therefore, the first therapeutic step should be using hydrolyzed formulas and, if symptoms persists, an amino acid-based formula [160]. Avoiding egg, soy, and wheat can be advised in a subset of patients with several FPE food triggers.

*Non-IgE-mediated Gastrointestinal Food Allergies: Conclusion*

Non-IgE-mediated gastrointestinal food-induced allergies are being increasingly diagnosed in children. The exact pathophysiology is still unclear and uniform evidence-based protocols are still lacking. The key to treatment of non-IgE-GIFA is eliminating the offending food(s), which should be done in collaboration with a dietician. Future studies are needed to further investigate pathogenesis, and to optimize management practices.

### 3.4. Eosinophilic Gastrointestinal Diseases

#### 3.4.1. Eosinophilic Esophagitis (EoE)

Evidence-based dietary recommendations:
  - Three different types of elimination diet can be used to induce remission: targeted elimination diet, empiric elimination diet and amino acid-based formula;
  - Empiric elimination diets include six-food elimination diet, four-food elimination, two-food elimination diet and one-food elimination diet;
  - Two possible approaches can be used: starting with a six-food elimination diet and performing one-by-one challenges (top-down approach) or starting with a two-food elimination diet and eventually eliminating others individual foods (step-up approach);
  - The elemental diet with amino acid-based formula is still a valid therapeutic choice in refractory disease.
False beliefs:
  - Positive skin prick tests or serum IgE tests can predict response to elimination of specific foods.

Overall, food avoidance seems to be more successful in pediatric patients than in adults in treating EoE [161]. However, determination of the causative foods of the disease can be challenging. 

Three different types of elimination diets have been assessed to induce remission in EoE: targeted elimination diet, empiric elimination diet and amino acid-based formula. 

The targeted exclusion diet consists of removing the food allergens most implicated with this disease. Unfortunately, since symptoms do not always correspond with mucosal inflammation, children need repeat endoscopies to assess for remission. Once remission is established, foods can be reintroduced to diet. However, the process of reintroduction and repeat endoscopy has not been universally established. Most studies failed to find clinical utility in the detection of triggers of EoE with blood IgE microarrays and skin prick and atopy patch [161,162,163,164,165,166]. However, targeted elimination of patch-test positive proteins may play a role in therapeutic management, as this strategy led to remission in 45% of patients [167]. The six most frequently implicated foods in EoE are milk, wheat, soy, and eggs (in order of frequency), followed by peanuts/nuts and fish/shellfish. Therefore, empiric elimination diets (removing these common allergens) have been introduced with the aim to overcome the low diagnostic accuracy of allergy tests and the unfeasibility of amino acid-based diets. The six-food elimination diet excludes the aforementioned six most frequently reported allergenic foods, is the best-studied and led to histological remission in 75% of patients [167]. Considering that the most common allergens, as assessed with single-food challenges, were CM, wheat, egg, and soy/legumes, the four-food elimination diet was introduced, with the advantage of being less restrictive and still achieving histological remission in 71% of patients [168]. Considering that animal milk and gluten-containing cereals are the two most frequent causative foods, a two-food elimination diet was also assessed [169]. In this European multicenter prospective study involving adults and children, the authors administered a two-food elimination diet for 6 weeks, achieving EoE remission in 43% of patients. In non-responders, a four-food elimination and six-food elimination diet were eventually offered, obtaining histological remission in 60 and 79%, respectively. There were no differences in achieving remission based on age of the patients. One-food elimination may have a role in treating infants and toddlers with EoE, considering that CM is the most common causative agent. A recent study performed on older children between 5 and 13 years of age demonstrated the one-food elimination diet induced histologic remission in 51% of children with EoE, suggesting its use as first-line therapy, being easily implemented [170]. This is a promising treatment option, but is not supported yet by randomized controlled trials [171].

In patients with several food allergies, severe failure to thrive, and/or refractory disease despite multiple elimination diets, an elemental diet with amino acid-based formula is a valid therapeutic option [172]. In fact, elemental diet was the first dietary intervention proposed to treat the symptoms of EoE in 1995 [173]. An elemental diet has an overall efficacy to induce histological remission of 90.8% [167]. However, feasibility of a long-term elemental diet is limited [174]. Moreover, there are some concerns about possible delayed speech development in young children on an elemental diet. Facial muscles may be underdeveloped as their functioning is stimulated by chewing [175]. 

Regardless of the type of elimination diet that is recommended, dietician guidance is always advised in order to guarantee adherence to the diet and to prevent nutritional deficits. There are also psychosocial implications around a diagnosis of EoE if a child chooses diet-based therapy. Undertaking a restrictive diet can result in stressful interactions around mealtime with stress and reduction in quality of life for children and caregivers [176,177].

#### 3.4.2. Other Eosinophilic Gastrointestinal Diseases (Eosinophilic Gastritis EG, Eosinophilic Gastroentieritis EGE, Eosinophilic Colitis EC) and Diet

Evidence-based dietary recommendations:
  - Elimination diet and/or corticosteroids should be used as first-line therapy for inducing remission in EG;
  - Elemental diet or six-food elimination diet might be valid options to reach remission in EGE, thereby decreasing the use of medications;
  - A hydrolyzed protein formula or amino acid-based formula can be indicated as first line treatment in infant with EC, since CM is the most common trigger.
False beliefs:
  - IgE microarrays, skin prick and atopy patch are useful to predict responsiveness to specific food elimination diet;
  - Dietary modifications are as effective in adults as in children with EC.

Elimination diet in EG can induce remission of both the symptoms and the histological abnormalities in a sub-group of patients. In one study, an elimination diet led to 80% clinical remission and 78% histological remission of EG. Elimination diets included elemental diet and exclusion of CM, eggs, gluten, soy, nuts, fish/shellfish, and red meat. However, in this study, only up to five patients for each dietary treatment arm underwent an endoscopic revaluation; therefore, the study was inconclusive [178]. In the same study, skin prick tests or serum analyses failed to predict response to elimination diet [178].

In EGE, a great number of empiric elimination diets (milk, cereals, egg, soy, seafood, and fruits) have been proposed. The correlation between triggering foods and disease is very hard to establish. For infants under the age of 1, CM-free diet has successfully controlled the disease [179]. 

In a cohort of adult patients with EGE, six-food elimination diet or amino acid-based formula for 6 weeks led to a notable improvement in the clinical picture, the histological and endoscopic findings, and the peripheral eosinophilia [180]. 

Despite the clinical benefit of dietary intervention published in literature, the absence of non-invasive modalities to evaluate the histologic improvement after food elimination [180] makes dietary treatment difficult to manage in practice. 

With regards to EC, few reports have been published on the effectiveness of elemental diet or dietary elimination in pediatric patients. Because CM protein is the most frequent trigger of EC in this group, a hydrolyzed protein or amino acid-based formula might be recommended as first-line therapy [181]. The evidence of positive or negative IgE tests failed to predict responsiveness to exclusion diet [182]. Conversely to what happens in children, dietary modifications are usually not effective in adults.

If used, dietary therapies should be performed under the guidance of a dietician. Once remission is achieved, each food should be reintroduced slowly, preferably from the least to the most allergenic food, and it should be followed by the histological demonstration of remission.

*Eosinophilic gastrointestinal diseases: Conclusions*

EGIDs are chronic diseases, which are treated with elimination diets and/or specific medications. Evidence on the best therapeutic approach is still lacking, with the exception of EoE, for which studies with greater samples have been published. Diet plays a pivotal role in EGIDs, however, treatment choice relies mostly on case reports and small case series. Furthermore, identifying a trigger food can be challenging and, because of the chronic nature of EGIDs, patients would need to follow very restricted diets for long periods of time, which can significantly impact their quality of life. 

## 4. Summary 

**Figure 1:**
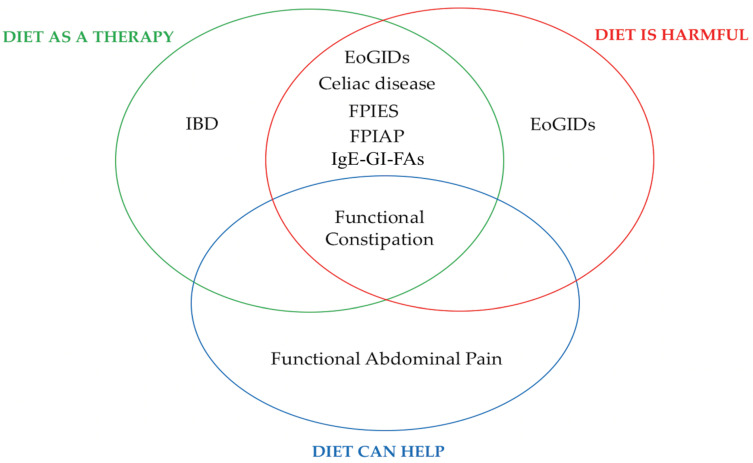
Summary of the multiple and complex nutritional aspect of pediatric gastrointestinal disorders.

Diets play multiple and complex roles in pediatric gastrointestinal disorders, as summarized in Figure 1. Foods might be the trigger for specific diseases such as in IgE or non-IgE-GIFAs, celiac disease, FPIES, or eosinophilic gastrointestinal diseases (EoGIDs). If a triggering food is clearly identified, the specific food elimination diet can be the best therapeutic choice for inducing remission. Nevertheless, when the incriminated food is not clearly identified, application of food-elimination strategies might be very challenging or too restrictive in the long term. Thus, drug-based therapies might be the better (or only) treatment.

On the other hand, diet might represent a therapeutic chance itself through adding specific foods/elements, which induce remission, e.g., the direct anti-inflammatory role of EEN in CD (whose function relies also in removing foods from diets); anti-inflammatory and antioxidant effects of curcumin in pediatric ulcerative colitis in addition to standard medication. 

Further, diets can help to improve gastrointestinal symptoms and might have an adjuvant role to medical therapies, such as reduced-FODMAP diet in IBS or fluid and fiber intake and probiotics/prebiotics in defecation disorders. Additionally, some patients with refractory chronic constipation might benefit from a trial of a pHF or CM-free diet.

## 5. Conclusions

In the last decade, the role of nutritional management in pediatric gastrointestinal diseases has gained increasing popularity. Apart from a diet’s therapeutic role, nutritional support is the crucial key in maintaining growth and improving clinical outcomes in pediatric patients. However, any dietary modification can have detrimental effects on the developing human body, whose growth relies on a balanced diet. Therefore, expert supervision is always advised.

In this current review, a global vision of the nutritional effects on gastrointestinal diseases is discussed. Advances and recent evidence linking nutrition to pediatric gastrointestinal diseases are reviewed, giving a practical guide for physicians and healthcare professionals.

## Figures and Tables

**Table 1:** Co-reactivity and food tolerance in food protein-induced enterocolitis syndrome (FPIES) [140,149,150,151].

Triggering Food	Co-Reactivity	Food Tolerance
CM	Soy (40–60%) Goat milk Sheep milk Any solid foods	Amino acid formula Extensively hydrolyzed formula * Baked CM *^,^° Milk from donkeys *^,^° Milk from camels *^,^° CM in maternal diet in breastfed infant (unless symptomatic) Food labelled “may contain traces of” *
Soy	CM (40%) Any solid food	
Rice	Oats (25–40%) Wheat (0–5%) Corn (1%)	
Egg		Baked eggs *^,^°
Fish	All fish (80%) Shellfish (50%)	Avoid all fish unless already tolerated
Chicken	All poultry (40%)	Avoid all poultry unless already tolerated
Any solid food	Other solid foods (44%)	

^*^ Might be tolerated; ° low level of evidence; to be administered with caution; Of note: Being tolerant to one food which belong to a category might predict tolerance to other foods belonging to that particular group.
